# Supplementary material for: Polyketide synthase gene clusters in the ericoid mycorrhizal fungus Oidiodendron maius: genome-wide prediction, expression profiling, and functional characterization of knockout mutants
Source: Front Plant Sci. 2026 Sep 16;17:1924430. doi: 10.3389/fpls.2026.1924430 (PMC13623903; doi:10.3389/fpls.2026.1924430)
Supplement: Supplementary file 1 [file SupplementaryFile1.zip › Supplementary Materials/Supplementary Folder 1.zip!/Oidma1_AssemblyScaffolds_Repeatmasked/knownclusterblast/cluster58/jgi.p_Oidma1_185052_mibig_hits.html]

| MIBiG Protein | Description | MIBiG Cluster | MiBiG Product | % ID | % Coverage | BLAST Score | E-value |
| --- | --- | --- | --- | --- | --- | --- | --- |
| AAK33069.1 | putative\_trichothecene\_biosynthetic\_gene | BGC0001278 | Nivalenol\_/\_deoxynivalenol\_/\_3-acetyldeoxynivalenol\_/ | 30 | 69.5374800638 | 183 | 7.6e-46 |
| AAK33079.1 | TRI10 | BGC0001277 | Nivalenol\_/\_deoxynivalenol\_/\_3-acetyldeoxynivalenol\_/ | 30 | 69.3779904306 | 182 | 1.7e-45 |
| AGO59048.1 | PtaR3 | BGC0000121 | Pestheic\_acid\_biosynthetic\_gene\_cluster | 29 | 76.2360446571 | 171 | 3e-42 |
| EED49427.1 | conserved\_hypothetical\_protein | BGC0000627 | Ustiloxin\_B\_biosynthetic\_gene\_cluster | 26 | 46.4114832536 | 80 | 7e-15 |
